# Clinical and electroencephalogram characteristics of pilomotor seizures

**DOI:** 10.1186/s42494-026-00260-8

**Published:** 2026-04-28

**Authors:** Zhijun Le, Bingqian Li, Hesheng Zhang, Yujie Chen, Raowei Yan, Dong Zhou, Jiani Chen, Xintong Wu

**Affiliations:** Department of Neurology, West China Hospital of Sichuan University, No. 37 Guoxue Alley, Wuhou District, Chengdu, 610041 Sichuan China

**Keywords:** Pilomotor seizures, Temporal lobe epilepsy, Ictal EEG, Autonomic manifestations

## Abstract

**Background:**

Piloerection, a physiological response to cold or emotional stimuli, is a rare autonomic manifestation of epileptic seizures. The anatomical correlates and electrophysiological mechanisms of pilomotor seizures remain poorly understood.

**Methods:**

We conducted a retrospective analysis of 13 patients with pilomotor seizures identified from 8482 individuals monitored at the Epilepsy Center of West China Hospital. Demographics, seizure characteristics, neuroimaging, and neurophysiological findings were analyzed to determine the epileptogenic zones and associated etiologies.

**Results:**

Most (11/13) of our cases showed temporal lobe origin of seizures, with distinctive ictal encephalogram patterns including rhythmic delta activity. Piloerection was consistently accompanied by other autonomic or psychic symptoms. Most patients responded to anti-seizure medications.

**Conclusions:**

Pilomotor seizures are associated with temporal lobe epilepsy and are frequently associated with specific etiologies such as autoimmune encephalitis.

## Background

Piloerection, commonly known as “goosebumps”, is a physiological phenomenon characterized by contraction of the arrector pili muscles in response to cold exposure or emotional stimuli [1]. Voluntary control of piloerection has been reported in a very small number of cases [2]. This suggests that this response is primarily mediated by the autonomic nervous system, with the hypothalamus, limbic system, and frontal cortex playing key roles in this process.

Piloerection is defined as an autonomic semiological feature of epileptic seizures in the International League Against Epilepsy (ILAE) 2025 position paper on updated classification of epileptic seizures [3]. In clinical practice, pilomotor seizures are often misdiagnosed as anxiety or depression when accompanied by emotional symptoms such as fear or other autonomic manifestations [4].

Given the infrequency of pilomotor seizures and the small number of video-EEG-documented cases, in this paper, we will summarize in detail the clinical features and epileptogenic zones of 13 cases of pilomotor seizures, in order to advance our understanding of this underrecognized semiology.

## Methods

Patients with epilepsy who underwent video-electroencephalogram (VEEG) monitoring at the Epilepsy Center of West China Hospital, Sichuan University, between January 2021 and October 2024, were screened. We retrospectively reviewed medical history data of these patients meeting the 2025 ILAE diagnostic criteria for epilepsy, to screen for the occurrence of piloerection [3]. Pilomotor seizures were identified based on synchronized video documentation or clinical confirmation of ictal manifestations by the medical team or family members, or based on patient self-report.

Patients with pilomotor seizures were enrolled in this study. Demographic data, semiological characteristics, and disease progression profiles were collected from these patients. Epileptogenic zones were evaluated based on these clinical parameters combined with multimodal neuroimaging and neurophysiological findings.

All patients underwent cranial magnetic resonance imaging (MRI), with varying scanning equipments and specific parameters. However, T1-weighted, T2-weighted, and FLAIR sequences were performed for all patients at a field strength of 3.0 T. The imaging features were independently evaluated by two neuroradiologists blinded to the patients' clinical information.

## Results

Among the 8482 patients diagnosed with epilepsy, 17 patients presented with piloerection as the ictal semiology. Four of them were excluded because of incomplete clinical data. Finally, a total of 13 right-handed patients (six males, seven females) were analyzed, with the mean age of 36.7 ± 10.9 years and the mean age at onset of 32.2 ± 12.7 years. The most common clinical diagnoses prior to admission included focal epilepsy of unknown etiology (*n*=10) and temporal lobe epilepsy (*n*=3). Ten patients experienced seizures during VEEG monitoring.

### Semiology

**Fig. 1:**
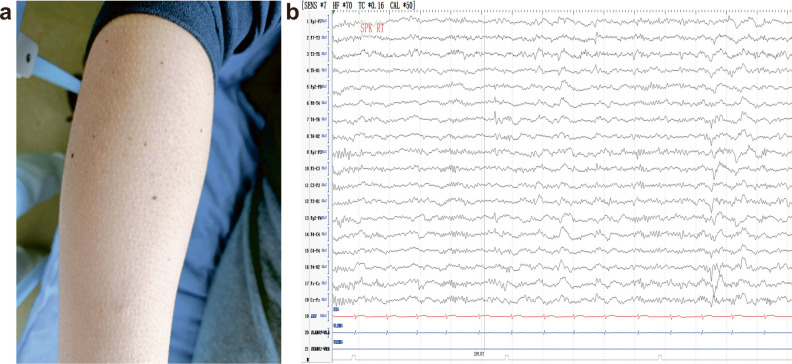
**a** A photo of piloerection in Case 1 during the seizure episode. **b** Interictal epileptiform discharges in the right temporal area of Case 6

In all the 13 cases, piloerection manifested as an integral component with other clinical presentation (Fig. 1a). Each patient demonstrated autonomic manifestations, including tachycardia, epigastric distress, nausea/vomiting, sweating, or olfactory hallucinations, déjà vu, limb paresthesia, altered consciousness, automatisms, or progression to generalized tonic-clonic seizures (Table 1). In particular, Case 7 exhibited cognitive problems, characterized by memory impairment, inability to recognize her way home, and lack of recall for recent events.

**Table 1:** Demographic and clinical characteristics of patients with pilomotor seizures

Case No.	Age (years)	Sex	Seizure frequency	Age of first seizure	Pilo onset region	Spreading	Semiology	IEDs	EEG onset	MRI	PET	SPECT	CSF autoantibodies	Etiology	Response to ASMs	Relevant medical history
1	36	M	0–5 per day	35	Right upper limbs	Whole body	Déjà vu, pilo, Right limbs numbness, olfactory hallucinations	Right temporal: sharp waves	Right temporal: 1–2 Hz slow wave→4–5 Hz rhythmic	Right temporal	Hypermetabolic in the right portion of the clivus	NA	Negative	Unclear	Good response to LCM	Nasopharyngeal carcinoma
2	46	F	3–4 per day	45	Bilateral limbs	Whole body	Increased heart rate, pilo, swallowing	Left temporal: sharp waves	Left frontal temporal: 2–3 Hz rhythmic slow wave	Left temporal	NA	NA	NA	Left temporal: cavernous angioma	Poor response to LEV, VPA	
3	33	F	2–3 per day	32	Left upper limbs	Left limbs	Left limb numbness, pilo	Right temporal: sharp waves	Right or left temporal: 4–5 Hz rhythmic→2–3 Hz rhythmic	Negative	NA	NA	NA	Unclear	NA, seizure free	NA
4	25	F	3–4 per week	16	Right limbs	Bilateral limbs	Chill, palpitation, pilo→swallowing/chewing, leftward gaze→asymmetric tonic posturing→GTCS	Middle line: SWC	Left frontal temporal: 2–3 Hz rhythmic slow wave	Left hippocampus sclerosis	Left hippocampus sclerosis; hypometabolic in left temporal; left FCD	Hyperperfusion in left temporal	NA	Left hippocampus sclerosis	Good response to left temporaloperation and LEV, ZNS	NA
5	34	F	1–2 per month	32	Right upper limbs	Whole body	Increased heart rate, auditory hallucinations, pilo→sweating	Left frontal temporal: sharp waves	Left temporal: 2–3 Hz slow waves→2–4 Hz SWC	Nodule in right temporal, probable cystic lesion	NA	NA	Negative	Unclear	Good response to PER	
6	36	M	3–4 per month	25	Right limbs	Whole body	Tachycardia, laughter, head deviation to the right, LOC→RUL flexion, RLL extension, L limbs hyperkinetic movements→lips pursing, left-hand automatisms→postictal aphasia	Bilateral temporal: sharp waves	Left frontal: rhythmic sharp waves	Negative	L F	NA	Negative	Left frontal: vascular malformation	Poor response to OXC, VPA, PER, LEV	
7	53	F	1–2 per month	47	Left upper limbs	Whole body	Precordial tightness, pilo	Right temporal: sharp waves	Right temporal: 3–4 Hz rhythmic slow wave→2–3 Hz SWC→2–3 Hz rhythmic slow waves	Bilateral Hipp and amygdala	Hypermetabolic in bi H and amygdala; hypometabolic in bi F	Hyperperfusion in the RTH, insula, basal ganglia region, thalamus and parieto-occipital junction region	LGI1	Autoimmune encephalitis	Poor response to LEV, LCM	
8	27	M	1–2 per month	25	Left face	No spreading	LOC, pilo	Bilateral temporal and bilateral Parietal: spikes and sharp waves	Right temporal: 2–3 Hz SWC	Left Hipp smaller than contralateral, normal signal	NA	NA	Negative	Lupus encephalitis?	Good response to LEV	Metabolic encephalopathy at 14; diagnosed with SLE at 20
9	44	M	2–3 per week	43	Bi face and bi upper limbs	No spreading	Optical hallucination, increased heart rate, pilo	No IEDs	Left temporal: 6–7 Hz slow waves→left frontal temporal:4–5 Hz slow waves	Negative	NA	NA	NA	Viral encephalitis	OXC	
10	25	M	3–4 per day	22	Right upper limbs	Bilateral limbs	Olfactory hallucinations,pilo, déjà vu, GTCS	No IEDs	No ictal EEG	Right temporal	NA	NA	Negative	Lupus encephalitis	Good response to LEV, LCM	Diagnosed with SLE at 23
11	34	F	1–2 per week	23	Bilateral	Whole body	Fear, pilo	Bilateral temporal: sharp waves	No ictal EEG	Negative	NA	NA	NA	Unclear	Good response to VPA, LCM	
12	25	F	3–4 per year	16	Unclear	Whole body	Fear, palpitations, chills, pilo, and rising epigastric sensation→repeated swallowing, tachycardia, tachypnea	Bilateral temporal: sharp waves and spike	Left temporal: 7–8 Hz rhythmic→3–5 Hz rhythmic	Bilateral hippocampal sclerosis	Bilateral hippocampal sclerosis	NA	Negative	Bilateral hippocampal sclerosis	Bilateral hippocampal sclerosis	Diagnosed with viral encephalitis at 16
13	59	M	1 per 2 days	57	Unclear	Face	Fear, pilo, GTCS	Right temporal: sharp waves	No ictal EEG	Right temporal	NA	NA	NA	Unclear	Good response to LCM, LEV	Craniotomy for sellar meningioma at 56

*ASMs* Anti-seizure medications, *CSF* Cerebrospinal fluid, *F* Female, *FCD* Focal cortical dysplasia, *GTCS* Generalized tonic-clonic seizures, *Hipp* Hippocampus, *IEDs* Interictal epileptiform discharges, *LCM* Lacosamide, *LEV* Levetiracetam, *LL* Lower limb, *LOC* Loss of consciousness, *M* Male, *MRI* Magnetic resonance imaging, *OXC* Oxcarbazepine, *PER* Perampanel, *PET* Positron emission tomography, *Pilo* Piloerection, *S* Sclerosis, *SLE* Systemic lupus erythematosus, *SPECT* Single-photon emission computed tomography, *SWC* Spike and wave complex, *UL* Upper limb, *VPA* Valproic acid, *ZNS* Zonisamide, *RUL* Right upper limb, *RLL* Right lower limb

“Good response” was defined as ≥ 50% reduction in seizure frequency or seizure freedom for at least 6 months; “poor response” was defined as < 50% reduction despite treatment with at least two appropriate ASMs

### EEG and imaging findings

Interictal epileptiform discharges (IEDs) were identified in 11 out of 13 patients (84.6%), predominantly (8 of 11) localized in the temporal region (four in the right, one in the left, and three in bilateral temporal lobe, Fig. 1b). The remaining three cases exhibited IEDs in the middle line (one case), the left frontotemporal region (one case), or the bilateral temporoparietal areas (one case).

**Fig. 2:**
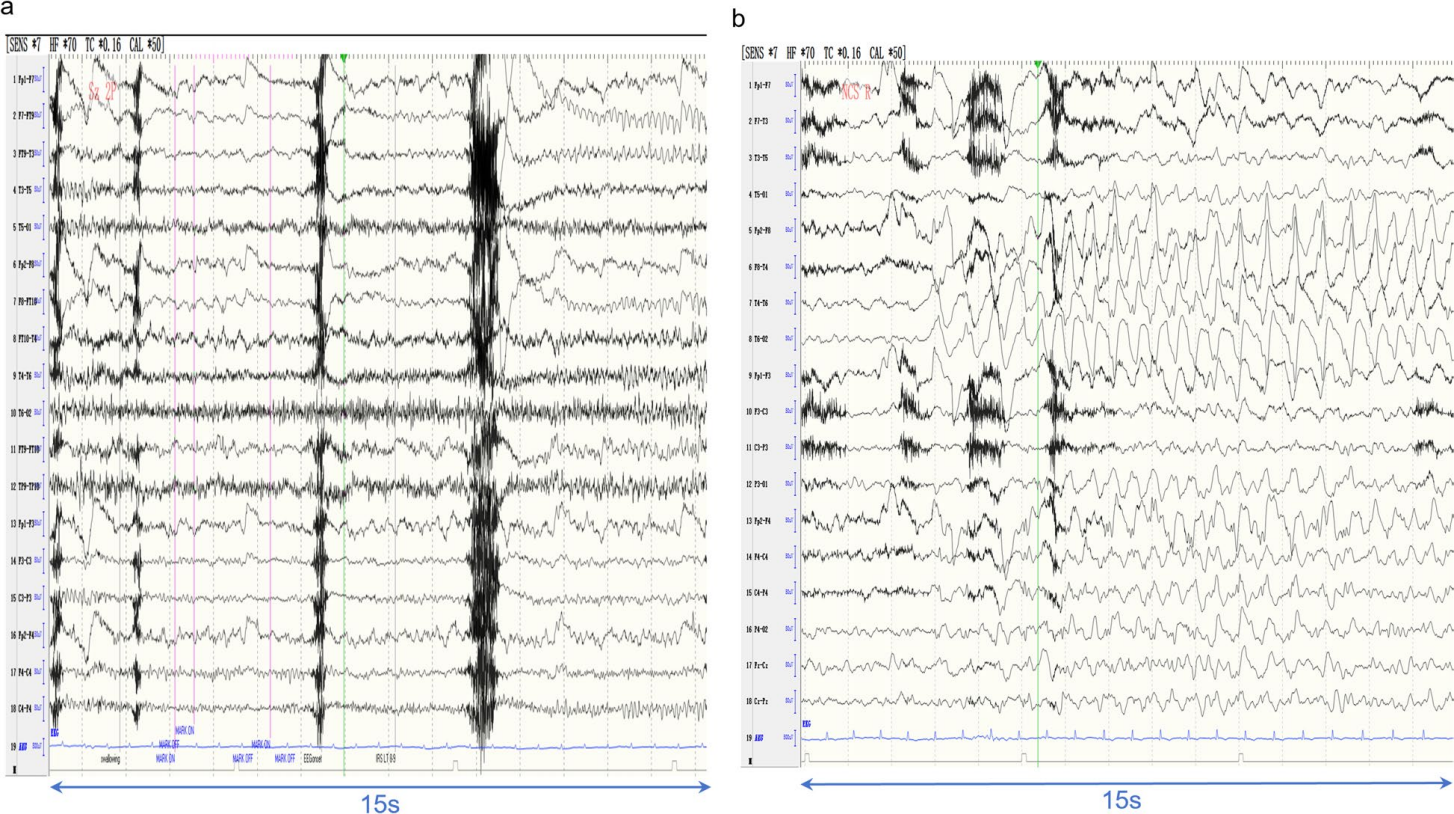
**a** In Case 12, the ictal EEG began as rhythmic 7–8 Hz activity, which subsequently evolved into 3–5 Hz discharges. **b** In Case 8, the ictal EEG recording during non-convulsive seizures showed 2–3 Hz spike-and-wave complex in the right hemisphere

**Fig. 3:**
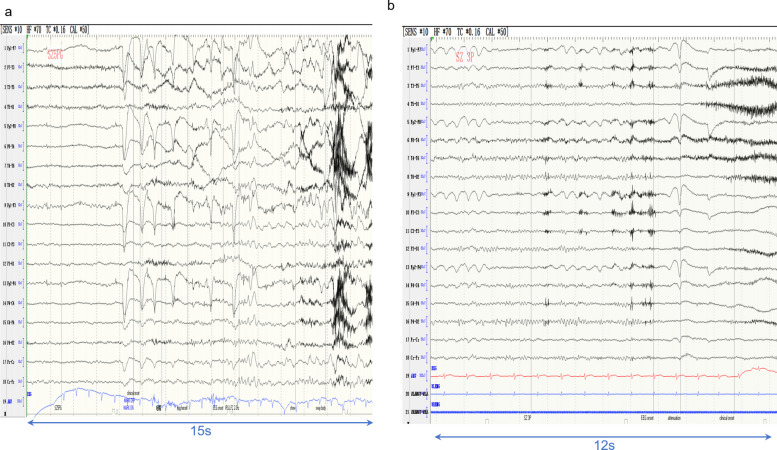
**a** Scalp ictal EEG of Case 4 demonstrated 2–3 Hz rhythmic slow-wave activity, a pattern also observed in Cases 1, 3, 5, 7, and 8. **b** Case 6 exhibited seizure onset with EEG activity originating from the frontocentral region

Ictal EEG recordings were successfully obtained from 10 patients. Seven cases showed temporal lobe origin of onset (three left-sided, Fig. 2a, three right-sided, Fig. 2b, and one bilateral), and three exhibited extra-temporal origin of onset, including the left frontotemporal region (two cases, Fig. 3a) and the left frontal lobe (one case, Fig. 3b).

**Fig. 4:**
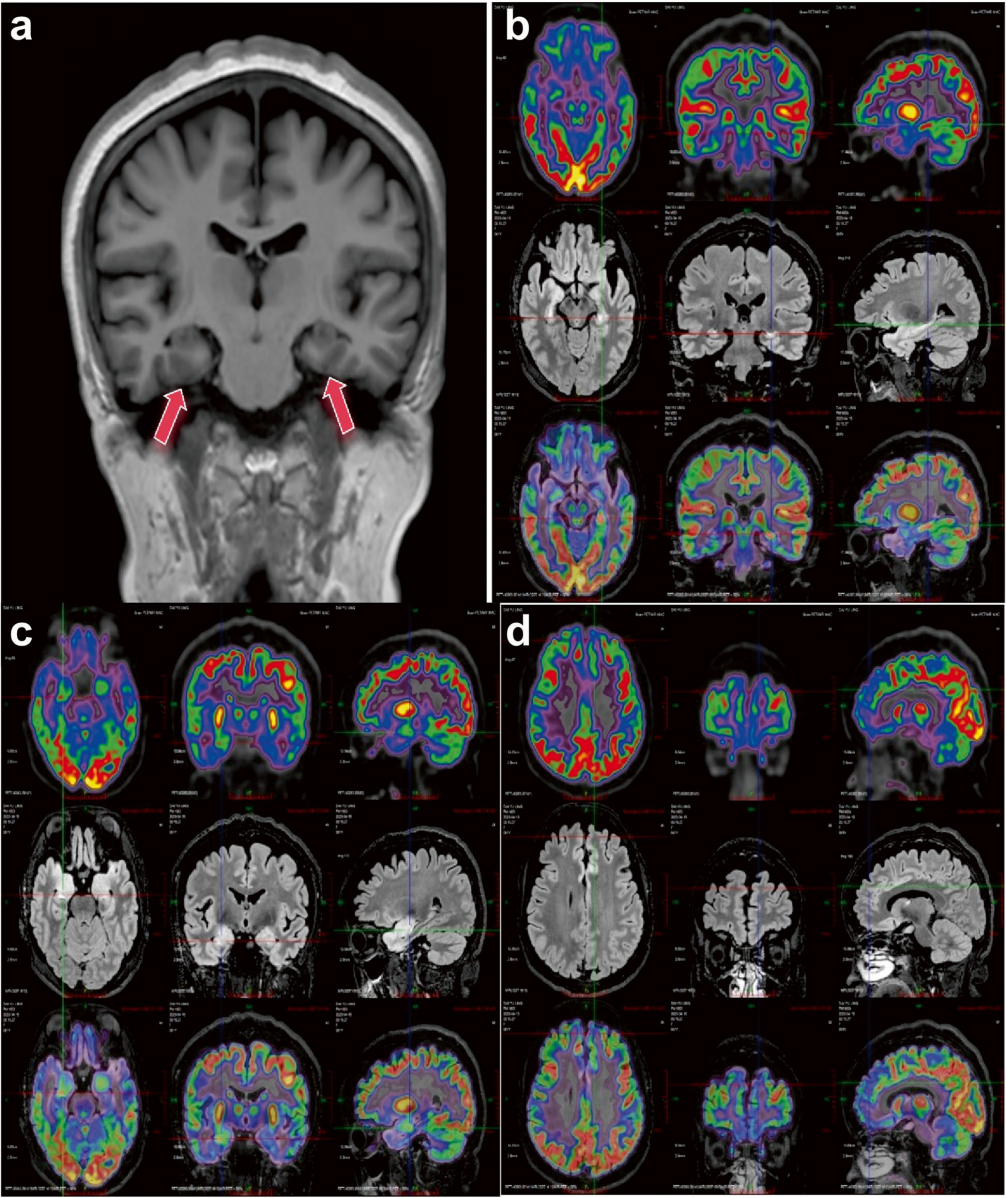
MRI and PET/MR images of Case 7. **a** MRI showed ill-defined, patchy hyperintensity in the bilateral hippocampi and amygdala on T1-weighted, suggesting encephalitis related changes or other pathology. **b**,**c** The bilateral hippocampi and amygdala with abnormal signals showed abnormally increased glucose metabolism, predominant on the left hemisphere (**b**); **d** Signal abnormality and symmetrically decreased glucose metabolism in the bilateral anterior medial frontal lobes. The above findings are suggestive of autoimmune encephalitis

MRI revealed abnormalities in the temporal lobe or the hippocampus in nine patients, while four patients demonstrated normal imaging findings. The MRI abnormalities included temporal lobe nodules in two cases, temporal lobe cavernous angioma in one case, hippocampal sclerosis in three cases, bilateral temporal lobe changes associated with autoimmune encephalitis in one case (Fig. 4), and non‑specific hippocampal signal abnormalities in two cases. In addition, seven cases showed concordance between imaging findings and EEG onset, three had negative imaging findings (two had ictal EEG, one had no ictal EEG), one case had negative EEG findings with MRI finding in right temporal, and two cases showed discordance between EEG onset and imaging findings.

The duration of ictal EEG discharges in the 10 patients with recorded seizures ranged from 10 s to 5 min. The onset of piloerection could be identified in six patients. Two patients exhibited piloerection with onset at 1–4 s before EEG onset, while the remaining four patients showed piloerection occurrence at 2–12 s after EEG onset.

### Etiology

Brain MRI identified structural changes in nine patients, but not all had a definitive seizure etiology. Among the four MRI-negative patients, one (Case 9) had viral encephalitis. Overall, a definite etiology was determined in eight patients: encephalitis (*n*=4), hippocampal sclerosis (*n*=2), cavernous hemangioma (*n*=1), and vascular malformation (*n*=1). The remaining five patients had undetermined etiologies despite comprehensive evaluation. 

### Treatment response

The mean follow-up duration was 18.6 months (range: 6–36 months). Nine patients achieved significant seizure control with anti-seizure medications (ASMs). One patient discontinued medication voluntarily but remained seizure-free during one year follow-up. Case 4 underwent left temporal epileptogenic focus resection with selective hippocampectomy and has been seizure-free for nine months postoperatively. Three patients showed a poor therapeutic response. Case 6 continues to experience 3–4 seizures monthly despite treatment with four ASMs. The cognitive function of Case 7 partially improved after treatment with intravenous immunoglobulin and methylprednisolone, but still experienced 1–2 seizures per month.

## Discussion

The pilomotor seizure is a rare autonomic symptom of focal epileptic seizures, which may be overlooked during diagnosis. Here, we reported 13 cases of pilomotor seizures to explore the origin and related networks of this disease.

In the skin, the sympathetic nervous system, the arrector pili muscle (originating from the mesenchymal layer), and the hair follicle (originating from the epithelial layer) form a functionally integrated tri-lineage unit. The arrector pili muscle is anchored to the bulge region of the hair follicle and receives direct sympathetic innervation, enabling coordinated piloerection [5]. The neural circuit of piloerection is triggered by temperature or emotional stimuli and involves multi-level integration of the central and autonomic nervous systems [6]. The temperature-triggered pathway begins with cutaneous cold receptors (e.g., TRPM8), which are activated by low temperatures and transmit signals via Aδ/C fibers to the spinal dorsal horn. These signals then ascend through the spinothalamic tract to the parabrachial nucleus in the brainstem, which further activates neurons in the hypothalamic preoptic area [7]. The emotion-triggered pathway originates in the amygdala, which receives emotional signals (e.g., fearful or moving) and relays them to the hypothalamic paraventricular nucleus for integration [8]. The two pathways ultimately converge as hypothalamic signals descend through the brainstem to the spinal intermediolateral column. Postganglionic sympathetic fibers release norepinephrine, which acts on α1-adrenergic receptors in the arrector pili muscles to induce piloerection [9]. This neural activity is also modulated by higher cortical regions, including the prefrontal cortex (PFC) and anterior cingulate cortex (ACC) [10, 11]. The ictal piloerection observed in pilomotor seizures may result from pathological hyperactivation of the sympathetic-cutaneous circuitry, the thalamus or amygdala, by epileptogenic networks.

In our case series, pilomotor seizures predominantly affected adults. Among the 13 cases, the mean age of first seizure was 32.2 years (range: 16–57), with 11 cases occurring at adulthood and 2 cases at 16 years. This age distribution may be caused by the fact that predominantly adult populations were treated at our epilepsy center. A previous literature review of 104 cases revealed that among 64 patients with documented age of onset (mean age of onset, 40.4 years; range: 1–82), 56 cases had seizure occurrence in adulthood. A few studies reported piloerection in underage epilepsy patients [12, 13]. In a retrospective study, 60 children with epilepsy exhibited autonomic symptoms, but none demonstrated piloerection [14]. These findings suggest that pilomotor seizures may be associated with neurological development and maturation.

Pilomotor seizures exhibit a strong association with temporal lobe origin. In our case series, 11 of 13 patients demonstrated temporal lobe origin. This was consistent with previously published case series. In a systematic review, Tényi et al. identified temporal lobe involvement in approximately 90% of well-localized cases. Similarly, Loddenkemper et al. reviewed 14 cases and reported temporal lobe origin in 85.7% of patients, close to the 84.6% observed in our series [15, 16]. Another study reported that approximately 10% of pilomotor seizures originate from extra-temporal regions, with frontal and parietal lobes being the most frequently involved [17].

In our study, among the nine patients with ictal EEG evidence of temporal onset, seven exhibited rhythmic delta activity (1–4 Hz)., which was distinctive from the characteristic theta rhythm typically observed in mesial temporal lobe epilepsy [18]. This discrepancy indicates a unique pathophysiological mechanism of pilomotor seizures and the value of precise localizing.

The value of pilomotor seizure lateralization is not fully understood. In our series of 13 cases, nine clearly exhibited unilateral onset of piloerection, including two patients showing strictly ipsilateral propagation and seven cases with contralateral spread. The initial side of piloerection was contralateral to the epileptogenic zone in six cases, and only one case showed ipsilateral correspondence. Previous studies have primarily focused on documenting the presence or distribution of piloerection, while few have systematically characterized initial localization and propagation patterns. Therefore, recording both the originating side and the spreading characteristics of pilomotor manifestations may provide valuable lateralizing information for the epileptogenic zone. However, given that epilepsy is a network disease, localizing the epileptogenic zone based solely on piloerection requires caution. However, the localizing value of this stereotyped clinical manifestation occurring at seizure onset should not be underestimated.

Pilomotor seizures may demonstrate significant associations with specific etiologies, particularly autoimmune encephalitis and nervous system tumors. In our series of 13 cases, one tested positive for LGI1 antibody, while two others had comorbid systemic lupus erythematosus. A systematic literature review of 109 cases identified 30 patients with confirmed autoimmune encephalitis, and approximately 46% of pilomotor seizure cases were associated with astrocytoma or limbic encephalitis [15]. Pilomotor seizures occur in about 0.65% of patients with refractory temporal lobe epilepsy [19], whereas the prevalence rises to 13.3–14% in limbic encephalitis populations [20–22]. These findings suggest that pilomotor seizures may be linked to immune-mediated neuroinflammatory processes.

The majority of patients with pilomotor seizures demonstrate favorable responses to ASMs, achieving adequate seizure control through standardized treatment protocols. For cases with identifiable etiology, a multimodal therapeutic approach is indicated.

Several limitations should be acknowledged. The retrospective design may introduce selection bias, and the number of included cases, while relatively large for this rare phenomenon, was modest. Additionally, some of the patients did not receive advanced brain imaging or comprehensive autoimmune testing, which may have limited the etiological classification. Future prospective, multicenter studies with standardized diagnostic protocols are warranted.

## Conclusions

Our study demonstrated that pilomotor seizures are associated with an origin in the temporal lobe. These seizures are characterized by specific ictal EEG patterns, notably rhythmic delta activity, and are almost invariably accompanied by other autonomic or psychic symptoms. Detailed documentation of the onset site and the propagation pattern of piloerection may provide valuable information on lateralization. Pilomotor seizures are frequently overlooked or misdiagnosed. Improved recognition and systematic characterization of this semiological feature are essential for accurate diagnosis, localization, and targeted management of epilepsy.

## Data Availability

The datasets generated in this study are available from the corresponding author upon reasonable request.

## Abbreviations

ACC: Anterior cingulate cortex
ASMs: Anti-seizure medications
CSF: Cerebrospinal fluid
EEG: Electroencephalogram
Fe: Female
FCD: Focal cortical dysplasia
GTCS: Generalized tonic-clonic seizures
Hipp: Hippocampus
IEDs: Interictal epileptiform discharges
ILAE: International League Against Epilepsy
LCM: Lacosamide
LEV: Levetiracetam
LL: Lower limb
LOC: Loss of Consciousness
M: Male
MRI: Magnetic resonance imaging
OXC: Oxcarbazepine
PER: Perampanel
PET: Positron emission tomography
PFC: Prefrontal cortex
Pilo: Piloerection
S: Sclerosis
SLE: Systemic lupus erythematosus
SPECT: Single-photon emission computed tomography
SWC: Spike and wave complex
UL: Upper limb
VEEG: Video-electroencephalogram
VPA: Valproic acid
ZNS: Zonisamide
